# Incidence and Predictors of Tuberculosis-associated IRIS in People With HIV Treated for Tuberculosis: Findings From Reflate TB2 Randomized Trial

**DOI:** 10.1093/ofid/ofae035

**Published:** 2024-01-22

**Authors:** Lara E Coelho, Corine Chazallon, Didier Laureillard, Rodrigo Escada, Jean-Baptiste N’takpe, Isabelle Timana, Eugène Messou, Serge Eholie, Celso Khosa, Giang D Chau, Sandra Wagner Cardoso, Valdiléa G Veloso, Constance Delaugerre, Jean-Michel Molina, Beatriz Grinsztejn, Olivier Marcy, Nathalie De Castro

**Affiliations:** National Institute of Infectious Diseases Evandro Chagas, Oswaldo Cruz Foundation, Rio de Janeiro, Brazil; National Institute for Health and Medical Research (INSERM) UMR 1219, Research Institute for Sustainable Development (IRD) EMR 271, Bordeaux Population Health Centre, University of Bordeaux, Bordeaux, France; Department of Infectious and Tropical Diseases, Nimes University Hospital, Nimes, France; Research Unit 1058, Pathogenesis and Control Chronical Infections, INSERM, French Blood Center, University of Montpellier, Montpellier, France; National Institute of Infectious Diseases Evandro Chagas, Oswaldo Cruz Foundation, Rio de Janeiro, Brazil; National Institute for Health and Medical Research (INSERM) UMR 1219, Research Institute for Sustainable Development (IRD) EMR 271, Bordeaux Population Health Centre, University of Bordeaux, Bordeaux, France; Programme PACCI/ANRS Research Center, Abidjan, Côte-d'Ivoire; Instituto Nacional de Saúde, Marracuene, Mozambique; Programme PACCI/ANRS Research Center, Abidjan, Côte-d'Ivoire; Centre de Prise en Charge de Recherche et de Formation, CePReF-Aconda-VS, Abidjan, Cote D'Ivoire; Département de Dermatologie et d'Infectiologie, UFR des Sciences Médicales, Université Félix Houphouët Boigny, Abidjan, Cote d'Ivoire; Programme PACCI/ANRS Research Center, Abidjan, Côte-d'Ivoire; Département de Dermatologie et d'Infectiologie, UFR des Sciences Médicales, Université Félix Houphouët Boigny, Abidjan, Cote d'Ivoire; Instituto Nacional de Saúde, Marracuene, Mozambique; Pham Ngoc Thach Hospital, Ho Chi Minh City, Vietnam; National Institute of Infectious Diseases Evandro Chagas, Oswaldo Cruz Foundation, Rio de Janeiro, Brazil; National Institute of Infectious Diseases Evandro Chagas, Oswaldo Cruz Foundation, Rio de Janeiro, Brazil; Virology department, APHP-Hôpital Saint-Louis, Paris, France; INSERM U944, Paris, France; Université Paris Cité, Paris, France; INSERM U944, Paris, France; Université Paris Cité, Paris, France; Infectious Diseases Department, AP-HP-Hôpital Saint-Louis Lariboisière, Paris, France; National Institute of Infectious Diseases Evandro Chagas, Oswaldo Cruz Foundation, Rio de Janeiro, Brazil; National Institute for Health and Medical Research (INSERM) UMR 1219, Research Institute for Sustainable Development (IRD) EMR 271, Bordeaux Population Health Centre, University of Bordeaux, Bordeaux, France; National Institute for Health and Medical Research (INSERM) UMR 1219, Research Institute for Sustainable Development (IRD) EMR 271, Bordeaux Population Health Centre, University of Bordeaux, Bordeaux, France; Infectious Diseases Department, AP-HP-Hôpital Saint-Louis Lariboisière, Paris, France

**Keywords:** antiretroviral therapy, HIV/AIDS, IRIS, randomized controlled trial, tuberculosis

## Abstract

**Background:**

After antiretroviral therapy (ART) initiation, people with HIV (PWH) treated for tuberculosis (TB) may develop TB-associated immune reconstitution inflammatory syndrome (TB-IRIS). Integrase inhibitors, by providing a faster HIV-RNA decline than efavirenz, might increase the risk for this complication. We sought to assess incidence and determinants of TB-IRIS in PWH with TB on raltegravir- or efavirenz-based ART.

**Methods:**

We conducted a secondary analysis of the Reflate TB 2 trial, which randomized ART-naive PWH on standard TB treatment, to receive raltegravir- or efavirenz-based ART. The primary objective was to evaluate the incidence of TB-IRIS. Incidence rate ratio comparing TB-IRIS incidence in each arm was calculated. Kaplan-Meier curves were used to compare TB-IRIS–free survival probabilities by ART arm. Cox regression models were fitted to analyze baseline characteristics associated with TB-IRIS.

**Results:**

Of 460 trial participants, 453 from Brazil, Côte d’Ivoire, Mozambique, and Vietnam were included in this analysis. Baseline characteristics were median age 35 years (interquartile range [IQR], 29–43), 40% female, 69% pulmonary TB only, median CD4, 102 (IQR, 38–239) cells/mm³, and median HIV RNA, 5.5 (IQR, 5.0–5.8) log copies/mL. Forty-eight participants developed TB-IRIS (incidence rate, 24.7/100 PY), 19 cases in the raltegravir arm and 29 in the efavirenz arm (incidence rate ratio 0.62, 95% confidence interval .35–1.10). Factors associated with TB-IRIS were: CD4 ≤ 100 cells/μL, HIV RNA ≥500 000 copies/mL, and extrapulmonary/disseminated TB.

**Conclusions:**

We did not demonstrate that raltegravir-based ART increased the incidence of TB-IRIS compared with efavirenz-based ART. Low CD4 counts, high HIV RNA, and extrapulmonary/disseminated TB at ART initiation were associated with TB-IRIS.

## Teaser

Integrase strand transfer inhibitor–based antiretroviral therapy (ART) did not increase the risk of tuberculosis-associated immune reconstitution inflammatory syndrome (TB-IRIS) in the Reflate TB2 randomized controlled trial. Low CD4 counts and high HIV-RNA at ART initiation and extrapulmonary/disseminated TB were risk factors for TB-IRIS.

Tuberculosis (TB) remains the leading cause of death in people with HIV (PWH). In 2021, of 660 000 deaths reported in PWH, 187 000 were due to TB [1]. In PWH treated for TB, antiretroviral therapy (ART) should be started after 2 weeks of TB treatment, especially in people with advanced HIV disease, and should include preferably the integrase strand transfer inhibitor (INSTI) dolutegravir [2].

A major concern when starting ART in PWH with TB is the occurrence of immune reconstitution inflammatory syndrome (IRIS) that can cause significant morbidity and sometimes death [3–5]. TB-IRIS can manifest as 2 different syndromes: paradoxical TB-IRIS, which is characterized by clinical and/or radiological worsening of TB manifestations after ART initiation, and unmasking TB-IRIS, in which a subclinical TB case is diagnosed by the emergence of clinically apparent signs and symptoms in the weeks following ART initiation [6]. TB-IRIS occurs in approximately 18% of PWH with TB but could affect up to 50% of those with very low CD4+ T-cell counts (<50/mm^3^), with disseminated TB, and a short interval between TB treatment and ART introduction [5]. The diagnosis of TB-IRIS is difficult in the absence of a confirmatory test. Several studies have documented increased levels of pro-inflammatory cytokines such as interleukin-6 and tumor necrosis factor-α during TB-IRIS but no definitive biomarker for clinical use has been yet established [7–9]. Clinical criteria have been developed by the International Network for the Study of HIV-associated IRIS (INSHI) to help clinicians diagnose TB-IRIS [6] but ruling out another HIV-related complication or resistance to TB treatment is almost impossible in many settings. Moreover, clinical management of most severe forms of IRIS can be challenging in resource-limited settings that have constrained access to invasive procedures such as drainage of compressive abscesses or ventriculoperitoneal shunt in TB meningitis cases [5, 10].

High levels of HIV-RNA and low CD4+ T-cell counts at ART initiation, rapid HIV-RNA decay, early ART introduction after TB treatment initiation (<2 weeks), and having extrapulmonary or disseminated TB have been identified as risk factors for TB-IRIS in observational studies and randomized controlled trials [4, 5, 11–13]. INSTI-based ART leads to faster decay of HIV-RNA levels than efavirenz-based ART [14]. This observation raised concerns about a potentially higher risk of TB-IRIS in PWH receiving INSTI-based regimen while being treated for TB, but data from randomized controlled trials are limited [3, 15].

In this substudy of the Reflate TB 2 trial, we aimed to assess whether the incidence of TB-IRIS was higher in PWH receiving raltegravir-based ART compared with those receiving efavirenz-based ART. In addition, we sought to identify determinants of TB-IRIS occurrence and describe TB-IRIS manifestations and outcomes.

## METHODS

### Study Design and Population

We conducted a secondary analysis of the ANRS 12 300 Reflate TB 2 trial. The design and study results have been reported previously [16, 17]. Between September 2015 and January 2018, ART-naïve adults with HIV-1 (age ≥18 years) with TB who had initiated TB treatment within the prior 8 weeks were randomized (1:1) to start ART with either raltegravir 400 mg twice daily or efavirenz 600 mg once daily, both in association with tenofovir disoproxil fumarate and lamivudine.

In the present study, we included all participants enrolled in the Reflate TB 2 trial in Brazil, Côte d’Ivoire, Mozambique, and Vietnam, excluding those with HIV-1 RNA < 50 copies/mL at baseline (n = 3) [16]. Participants enrolled in France (n = 4) were also excluded to restrict our analysis to low- and middle-income countries.

### Patient Consent Statement

The Reflate TB2 study protocol was approved by relevant national and local ethics committees in all participating countries and registered with ClinicalTrials.gov (NCT02273765). All participants provided written informed consent before enrollment in the main trial.

### Study Procedures

All participants received a standard TB treatment regimen, with 2 months of intensive phase (isoniazid [4–6 mg/kg/day], rifampicin [8–12 mg/kg/day], pyrazinamide [20–30 mg/kg/day], and ethambutol [15–20 mg/kg/day]) followed by 4 months of maintenance phase (isoniazid [4–6 mg/kg/day] and rifampicin [8–12 mg/kg/day]). After enrollment, clinical and laboratory assessments were performed at weeks 2, 4, 8, 12, 16, 20, 24, 32, 40, and 48.

Results of smear microscopy, Xpert MTB/RIF (Cepheid, Sunnyvale, CA, USA), mycobacterial cultures on expectorated sputum and relevant extrapulmonary samples, and chest X-rays were obtained at inclusion in the trial. During follow-up, sputum smear microscopy and culture and chest X-ray were done at baseline, week 8, and at the end of TB treatment. HIV-1 RNA levels were measured at baseline, weeks 4, 12, 24, 32, 40, and 48; CD4+ T-cell counts were obtained at baseline and weeks 4, 12, 24, and 48. Adherence to ART was estimated using pill count adherence ratio calculated from baseline to week 24 and from baseline to week 48.

During the trial, if an IRIS event was suspected, a detailed assessment was conducted at the site and all data relevant to the event were recorded in a specific case report form (IRIS suspicion form, Supplementary material). This form collected standardized data on first symptom onset date, clinical manifestations (fever, peripheral lymph nodes, central nervous system disorders), imaging features (chest X-ray, abdominal ultrasound, computed tomography scan), and IRIS treatment and management (steroids use, nonsteroidal anti-inflammatory drugs [NSAIDs] use, drainage, surgical procedures). IRIS clinical management was left at the discretion of the local investigators. All IRIS cases were then reviewed by countries’ endpoint review committee (ERC), each consisting of (1) the country principal investigator; (2) 1 or several other adequately trained physicians, selected by the country principal investigator; and (3) a representative of the country clinical trial unit.

### Outcomes and Definitions

The primary outcome of the study was the occurrence of paradoxical TB-IRIS in the Reflate TB2 trial. Paradoxical TB-IRIS was defined according to the INSHI criteria [6], detailed in Supplementary material. Secondary study outcomes included TB-IRIS manifestations (clinical and imaging features) and outcomes (TB-IRIS complete resolution without sequelae, hospitalization, events warranting steroid use or drainage, events that resulted on ART or TB treatment interruptions and death). We have also assessed the proportion of participants with HIV-RNA < 50 copies/mL at week 4, 12, 24, and 48 in TB-IRIS and non–TB-IRIS groups and the proportion of participants with optimal adherence to ART (pill count adherence ratio ≥ 95%) in both TB-IRIS and non–TB-IRIS groups.

All TB-IRIS reported by local investigators was initially reviewed by countries’ ERC and included in the study dataset using standardized forms. For this study, all IRIS cases reported by the investigators were further reviewed by a central ERC (L.E.C., N.D.C., R.E., S.W.C., O.M., D.L.) masked for ART treatment arm. The central ERC classified IRIS cases as TB-IRIS or non–TB-IRIS; only TB-IRIS cases underwent validation according to INSHI criteria. Moreover, reports of new signs and symptoms suggestive of TB-IRIS during the trial (ie, lymphadenopathy, constitutional symptoms, respiratory symptoms, abdominal pain, serositis, neurological symptoms) and use of steroids or NSAIDs up to 6 months after ART initiation were also reviewed by the central ERC to identify potential unreported TB-IRIS cases.

### Statistical Analysis

We described characteristics in each group (“TB-IRIS” and “no TB-IRIS”) using frequency and proportions for qualitative variables, and median and interquartile range (IQR) for quantitative variables. We compared characteristics between groups using Wilcoxon tests (comparison of medians) for quantitative variables, and the χ^2^ or the Fisher exact tests for qualitative variables, as appropriate. TB-IRIS manifestation and outcome absolute numbers and frequencies were estimated.

Incidence of TB-IRIS was estimated by 100 person-years with respective 95% exact Poisson confidence interval (CI). Participants’ follow-up time started on the day of the ART initiation and ended at the date of TB-IRIS onset for those who developed TB-IRIS. For participants without TB-IRIS, follow-up ended (censor date) at the date of death, date of last study visit, or 6 months after ART initiation, whichever occurred first. Kaplan-Meier curves with log-rank tests were used to compare TB-IRIS–free survival probabilities by ART treatment arm (raltegravir vs efavirenz). The incidence rate ratio comparing TB-IRIS incidence in each arm, along with 95% CIs and *P* values were also calculated. Cox regression models were used to assess baseline factors associated with TB-IRIS incidence. We selected the following variables based on previous evidence from the literature and clinical relevance: country, baseline CD4 T-cell counts and HIV-1 RNA, and type of TB disease at baseline (pulmonary only vs extrapulmonary, with or without concomitant pulmonary TB). The final multivariable model was reached using a stepwise selection procedure, retaining all variables with a level of significance (*P* = .05). This procedure modifies the forward selection technique by allowing effects already in the model to be removed. Proportional hazards assumption was tested graphically. All statistical analysis was performed using SAS software (version 9.4 M3).

## RESULTS

**Figure 1.**
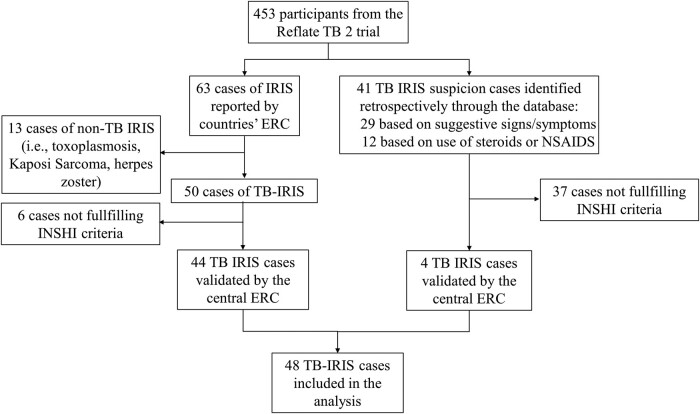
Study flow chart. ERC, endpoint review committee; INSHI, International Network for the Study of HIV-associated IRIS; IRIS, immune reconstitution inflammatory syndrome; NSAIDs, nonsteroidal anti-inflammatory drugs; TB, tuberculosis.

Of the 460 participants enrolled in the Reflate TB 2 trial, 453 were included in the present analysis (Figure 1). Study participants had advanced HIV disease, with a median CD4+ T-cell count of 102 (IQR, 38–239)/mm³ and median HIV-1 RNA of 5.5 (IQR, 5.0–5.8) log_10_ copies/mL, with 74.9% (337/450) of participants with HIV-1 RNA ≥100 000 copies/mL (Table 1). Overall, participants started ART at a median of 20 (IQR, 15–27) days after TB treatment initiation.

**Table 1.** Participant Characteristics by TB-IRIS Occurrence

	N^a^	TB-IRIS	N^a^	No TB-IRIS	N^a^	Total	*P*
		N = 48		N = 405		N = 453	
Antiretroviral treatment, n (%)		…	…	…	…	…	.12
Efavirenz	…	29 (60.4)	…	196 (48.4)	…	225 (49.7)	
Raltegravir	…	19 (39.6)	…	209 (51.6)	…	228 (50.3)	
Country, n (%)	…	…	…	…	…	…	.009
Ivory Coast	…	17 (35.4)	…	153 (37.8)	…	170 (37.5)	
Brazil	…	5 (10.4)	…	38 (9.4)	…	43 (9.5)	
Vietnam	…	20 (41.7)	…	91 (22.5)	…	111 (24.5)	
Mozambique	…	6 (12.5)	…	123 (30.4)	…	129 (28.5)	
Baseline variables							
Age, y	…	33.4 (29.0–42.8)	…	35.3 (28.9–42.9)	…	35.3 (28.9–42.9)	1.00
Female sex, n (%)	…	15 (31.3)	…	166 (41.0)	…	181 (40.0)	.19
BMI (kg/m^2^), median (IQR)	47	17.9 (16.9–19.8)	404	19.2 (17.6–21.2)	451	19.1 (17.5–21.0)	.007
Hemoglobin (g/dL), median (IQR)	…	10.0 (8.2–11.1)	…	9.8 (8.5–11.3)	…	9.8 (8.5–11.3)	.54
CD4+ T-cell counts (cells/mm^3^)							
Median (IQR)	…	55.0 (25.0–98.5)	404	114.5 (42.0–249.5)	452	102.0 (37.5–238.5)	<.001
≤ 50, n (%)	…	22 (45.8)	404	130 (32.2)	452	152 (33.6)	<.001
51–100, n (%)	…	14 (29.2)	404	58 (14.4)	452	72 (15.9)	
101–200, n (%)	…	8 (16.7)	404	78 (19.3)	452	86 (19.0)	
> 200, n (%)	…	4 (8.3)	404	138 (34.2)	452	142 (31.4)	
HIV-1 RNA (copies/mL)							
Log_10_, median (IQR)	47	5.8 (5.5–6.1)	403	5.4 (5.0–5.8)	450	5.5 (5.0–5.8)	< .001
< 100,000, n (%)	47	2 (4.3)	403	111 (27.5)	450	113 (25.1)	< .001
100 000–499,999, n (%)	47	15 (31.9)	403	166 (41.2)	450	181 (40.2)	
≥ 500,000, n(%)	47	30 (63.8)	403	126 (31.3)	450	156 (34.7)	
TB anatomical site at baseline, n (%)							
Pulmonary only	…	25 (52.1)	…	287 (70.9)	…	312 (68.9)	.008
Extrapulmonary with or without concomitant pulmonary TB	…	23 (47.9)	…	118 (29.1)	…	141 (31.1)	
Bacteriologically confirmed TB, n (%)	47	37 (78.7)	401	270 (67.3)	448	307 (68.5)	.11
Smear microscopy positive for acid-fast bacilli	47	23 (48.9)	397	183 (46.1)	444	206 (46.4)	.71
Xpert MTB/RIF test positive	46	29 (63.0)	384	237 (61.7)	430	266 (61.9)	.86
Culture positive for *Mycobacterium tuberculosis*	47	27 (57.4)	391	199 (50.9)	438	226 (51.6)	.40
Days on TB treatment at ART initiation, median (IQR)	…	20.5 (15.0–29.0)	…	20.0 (15.0–27.0)	…	20.0 (15.0–27.0)	0. 75
Laboratorial results during follow-up							
Plasma HIV-1 RNA < 50, n (%)							
At week 4	42	2 (4.8)	396	63 (15.9)	438	65 (14.8)	.03
At week 12	43	10 (23.3)	378	206 (54.5)	421	216 (51.3)	<.001
At week 24	…	21 (43.8)	…	247 (61.0)	…	268 (59.2)	.02
At week 48	…	25 (52.1)	…	264 (65.2)	…	289 (63.8)	.07
HIV-1 RNA variation from baseline (log 10), median (IQR)							
At week 4	41	−2.9 (−3.4 - −2.3)	394	−2.8 (−3.3 - −2.3)	435	−2.8 (−3.3 - −2.3)	.99
At week 12	42	−3.6 (−4.1 - −1.9)	376	−3.6 (−4.0 - −3.0)	418	−3.6 (−4.0 - −3.0)	.70
CD4 variation from baseline (/mm³), median (IQR)							
At week 4	41	70.0 (37.0–117.0)	395	84.0 (21.0–152.0)	436	81.0 (24.0–149.5)	.72
At week 12	43	129.0 (40.0–184.0)	378	111.0 (48.0–196.0)	421	113.0 (47.0–193.0)	.79
ART adherence during follow-up							
Pill count adherence ratio W0-W24 ≥ 95%	…	32 (71.1)	…	289 (72.4)	…	321 (72.3)	.44
TB treatment outcome							
Cure: bacteriologically confirmed PTB smear and culture negative twice	…	23 (47.9)	…	209 (51.7)	…	232 (51.3)	.62
Treatment completed: treatment completed and no criteria for cure or failure	…	16 (33.3)	…	161 (39.9)	…	177 (39.2)	.38
Treatment failure: smear or culture positive at 5 mo or later	…	1 (2.1)	…	5 (1.2)	…	6 (1.3)	
Died for any reason during TB treatment^b^	…	6 (12.5)	404	16 (4.0)	452	22 (4.9)	

Data are n (%) or median (IQR).

ART, antiretroviral therapy; BMI, body mass index; IQR, interquartile range; TB, tuberculosis; TB-IRIS, tuberculosis-associated immune reconstitution inflammatory syndrome; W0, week 0; W24, week 24.

^a^N when missing data.

^b^22 of the 26 persons who died during the 48 weeks of follow-up in the Reflate TB2 trial died before the end of TB treatment. Overall causes of death in the trial up to W48 are reported in Supplementary Table 1.

At enrollment, most of the participants (68.9%) had pulmonary TB, 56 (12.4%) had both extrapulmonary and pulmonary TB, and 85 (18.8%) had extrapulmonary TB only. The most common extrapulmonary manifestations were peripheral lymphadenopathy (12.8%), pleural effusion (10.8%), retroperitoneal lymphadenopathy (7.5%), and hilar/mediastinal lymphadenopathy (2.4%).

### TB-IRIS Incidence

Forty-eight participants (10.6%) developed TB-IRIS during follow-up, 19/228 (8.3%) participants in the raltegravir arm and 29/225 (12.9%) participants in the efavirenz arm, yielding an overall incidence rate of 24.7 per 100 person-years (95% CI, 18.2–32.7) and an incidence rate ratio of 0.62 (95% CI, .35–1.10; *P* = .10). The median time between ART introduction and TB-IRIS onset was 4.0 (IQR, 2.0–7.5) days.

Several baseline characteristics differed between participants with and without TB-IRIS. Participants who developed TB-IRIS had lower body mass index, lower CD4+ T-cell counts, higher plasma HIV-1 RNA, and higher frequency of extrapulmonary TB with or without concomitant pulmonary TB (Table 1). Among participants with baseline CD4+ T-cell count ≤ 50/mm³, 14.5% developed IRIS-TB (22/152), whereas among those with baseline CD4+ T-cell count > 200/mm³, only 2.8% developed IRIS-TB (4/142). There was no difference between the groups in the delay between TB treatment and ART initiation (median, 20.5 [IQR, 15.0–29.0] and median, 20.0 [IQR, 15.0–27.0] days, for TB-IRIS and no TB-IRIS, respectively). Moreover, delay between TB treatment and ART initiation were similar in participants with baseline CD4+ T-cell count ≤ 50/mm³ and those with CD4+ T-cell count >50/mm³ (*P* = .47).

### TB-IRIS Manifestations and Outcomes

Fever was the most common symptom (50.0%, 22/44), followed by peripheral lymphadenopathy (new or worsening, 40.9%, 18/44). New or worsening imaging features were found in 59.5% (22/37) of the participants with a chest X-ray available during TB-IRIS investigation and in 60% (18/30) of those who had an abdominal ultrasound performed during TB-IRIS investigation (Table 2).

**Table 2.** Clinical and Imaging Features of TB-IRIS Cases

	N = 48
Time between TB-IRIS and TB treatment initiation (wk), median (IQR)	7.4 (4.9–10.4)
Time between TB-IRIS and ART initiation (wk), median (IQR)	4.0 (2.0–7.5)
Clinical manifestations	
Fever	22/44 (50.0)
Peripheral lymph nodes (new or worsening)	18/44 (40.9)
CNS disorders	4/43 (9.3)
Abdominal pain	13/44 (6.8)
Respiratory symptoms	13/44 (6.8)
New or worsening imaging features	
Chest radiography	22/44 (50.0)
Chest radiography not performed	7/44 (15.9)
Ultrasound	18/44 (40.9)
Ultrasound not performed	14/44 (31.8)
Computed tomography scan	7/44 (15.9)
Computed tomography scan not performed	23/44 (52.3)
TB-IRIS outcomes	
TB-IRIS treatment with steroids	27/44 (61.4)
Abscess drainage	4/44 (9.1)
ART interruption because of TB-IRIS	1/44
TB treatment interruption because of TB-IRIS	0
Complete resolution of IRIS without sequelae	30/44 (68.2)
Hospitalization at the time of TB-IRIS^a^	9/44 (20.4)
Death related to TB-IRIS	2/44 (4.5)

Data are n (%) or median (IQR).

ART, antiretroviral therapy; CNS, central nervous system; IQR, interquartile range; TB, tuberculosis; TB-IRIS, tuberculosis-associated immune reconstitution inflammatory syndrome.

^a^Hospitalization occurred within 7 days of start of the onset of IRIS-TB symptoms.

Steroid treatment was initiated for 61.4% (27/44) of participants with TB-IRIS and 9.1% (4/44) of the participants underwent abscess drainage. ART treatment was temporarily interrupted in 2.1% (1/48) of the participants. There was no TB treatment interruption. Nine (18.8%) participants with TB-IRIS were hospitalized at the time of IRIS diagnosis (within 7 days of the onset of the symptoms) and 6 (12.5%) died. In 2 participants, death was deemed associated with TB and occurred at the time of IRIS occurrence, with no alternative diagnosis (Supplementary Table 1).

### Virologic Outcomes and Adherence

Relative to baseline levels, decays in HIV RNA measured at week 4 and at week 12 (absolute difference between week 4 or week 12 and baseline levels in log10 copies/mL) were not different between those who developed TB-IRIS and those without TB-IRIS (Table 1). Overall, 59.2% and 63.8% of the participants achieved HIV-RNA < 50 copies/mL at weeks 24 and 48, respectively. At both time points, these proportions were lower among those who developed TB-IRIS (43.8% and 52.1%) than those without TB-IRIS (61% and 65.2%) (Table 1). Similarly, at weeks 4 and 12, the proportion of participants with HIV-RNA < 50 copies/mL was significantly lower among those with TB-IRIS (4.8% and 23.3%, respectively) compared with those without TB-IRIS (15.9% and 54.5%, respectively). Adherence to ART (pill count adherence ratio ≥ 95%) within the first 24 weeks of the trial was similar in both groups: 32/45 (71.1%) participants with TB-IRIS and 289/299 (72.4%) participants without TB-IRIS.

### Factors Associated With TB-IRIS

**Figure 2.**
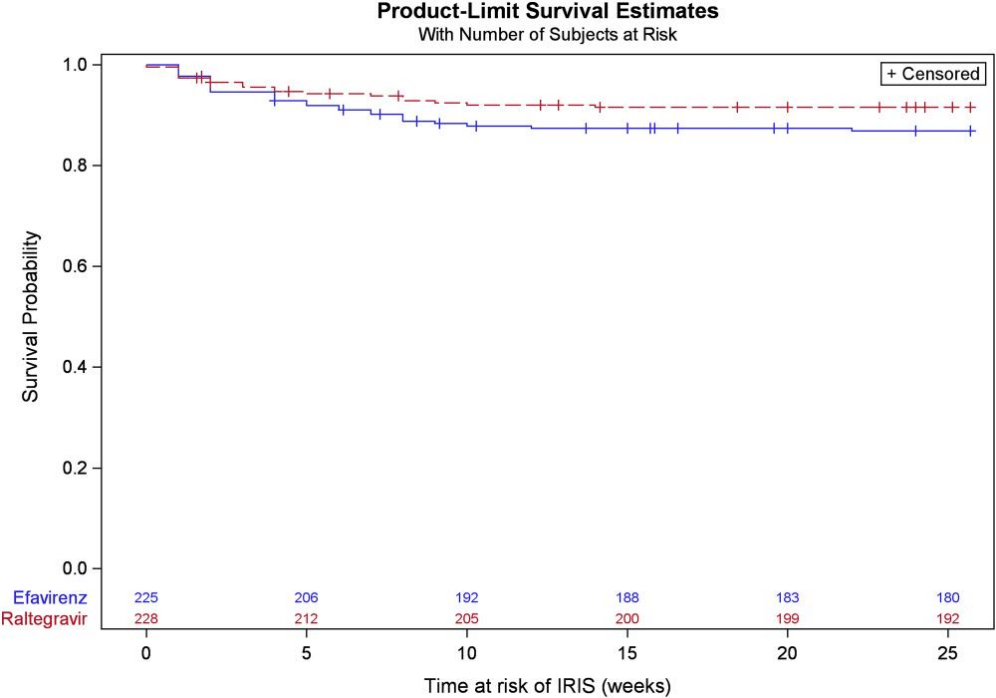
TB-IRIS free survival probabilities by ART treatment arm. ART, antiretroviral therapy; TB-IRIS, tuberculosis-associated immune reconstitution inflammatory syndrome; Log-rank *P* = .12.

ART regimen (raltegravir vs efavirenz) was not associated with the risk of developing TB-IRIS (Figure 2, log rank *P* = .12).

In the multivariable analysis, CD4+ T-cell count ≤100/mm³ (adjusted hazard ratio [aHR], 2.480; 95% CI, 1.269–4.846; reference > 100/mm³), HIV-1 RNA ≥500 000 copies/mL (aHR, 2.917; 95% CI, 1.588–5.358), and having “extrapulmonary TB with or without concomitant pulmonary TB” (aHR, 2.173; 95% CI, 1.226–3.851) were independently associated with TB-IRIS (Table 3).

**Table 3.** Factors Associated With TB-IRIS Incidence

	Total	TB-IRIS	Univariable Analysis			Multivariable Analysis				
	N	n (%N)	HR	Lower 95% CI	Upper 95% CI	Pr > χ2	HR	Lower 95% CI	Upper 95% CI	Pr > χ^2^
Country	…	…	…	…	…	.0130	…	…	…	…
Brazil	43	5 (11.6%)	1	…	…	…	…	…	…	…
Ivory Coast	170	17 (10.0%)	0.837	.307	2.286	…	…	…	…	…
Mozambique	129	6 (4.7%)	0.391	.119	1.282	…	…	…	…	…
Vietnam	111	20 (18.0%)	1.653	.620	4.404	…	…	…	…	…
CD4+ T-cell counts (cells/mm³)			…	…	…	0.002	…	…	…	0.008
>100	228	12 (5.3%)	1	…	…	…	…	…	…	…
≤ 100	224	36 (16%)	3.174	1.647	6.115	…	2.480	1.269	4.846	…
HIV-1 RNA (copies/mL)			…	…	…	<0.001	…	…	…	<0.001
< 500 000	294	17 (5.8%)	1	…	…	…	…	…	…	…
≥ 500 000	156	30 (19.2%)	3.649	2.012	6.617	…	2.917	1.588	5.358	…
TB anatomical site at baseline			…	…	…	0.006	…	…	…	0.008
Pulmonary only	312	25 (8%)	1	…	…	…	…	…	…	…
Extrapulmonary with or without concomitant pulmonary TB	141	23 (16%)	2.220	1.253	3.934	…	2.173	1.226	3.851	…

CI, confidence interval; HR, hazard ratio; TB, tuberculosis; TB-IRIS, tuberculosis-associated immune reconstitution inflammatory syndrome.

## DISCUSSION

In this secondary analysis from the Reflate TB 2 trial, we found a similar incidence of TB-IRIS in participants receiving raltegravir-based ART compared with those receiving efavirenz-based ART. In addition, we showed that low CD4+ T-cell counts, high HIV-1 RNA at ART initiation, and having extrapulmonary or disseminated TB were independently associated with TB-IRIS, which is consistent with previous studies [4, 5, 11–13].

Previous cohort studies had suggested that INSTI-based regimens were associated with increased risk of IRIS [3, 15]. In the Dat’AIDS prospective multicenter cohort in France, PWH with CD4+ T-cell counts below 200/mm^3^ (median, 83 cells/mm³) initiating an INSTI-based ART regimen had a 2-fold higher risk of all cause IRIS requiring hospitalization, relative to participants who initiated other ART regimens [3]. However, this association was not confirmed in randomized clinical trials [18]. Differences found between observational studies and randomized clinical trials may result from selection bias that often impact effect estimates in observational studies. The INSPRING trial, evaluated dolutegravir and efavirenz-based ART in PWH treated for TB and randomized 113 participants with CD4+ T-cell counts ≥ 50/mm³ (median, 208 cells/mm³) to receive either dolutegravir- (n = 69) or efavirenz- (n = 44) based ART [19]. Importantly, individuals with previous history of TB, those with central nervous system, miliary, or pericardial TB, as well as those with hepatic impairment at screening were excluded from the study. Similar to our results, they found that TB-IRIS incidence was low (7% [8/113] of the participants) and similar in both ART regimens; however, participants at higher risk of TB-IRIS were excluded from the study. Reflate TB2 randomized a significantly larger population (n = 460) with more advanced HIV and TB diseases. Indeed, median CD4+ T-cell counts was 104 cells, almost two thirds of the participants had CD4+ T-cell counts < 50 cells/mm^3^ and disseminated or extrapulmonary TB, and 35% had HIV RNA ≥ 500 000 copies/mL [16]. Taken together, the Reflate TB2 and INSPRING studies, despite very different study populations, provide evidence that INSTI-based regimens do not increase the risk of TB-IRIS. This is a reassuring finding in light of the current recommendation to use dolutegravir as first-line ART regimen in high-burden TB settings.

In our study, most of the TB-IRIS cases were not severe. Fever, lymphadenopathy, and respiratory symptoms were the most common manifestations, although half of the cases warranted steroids treatment and few cases required invasive procedures (drainage) but there were two deaths suspected to be related to TB-IRIS, one in raltegravir arm and one in the efavirenz arm. Lymphadenopathy, fever, and respiratory symptoms have been described as most common clinical manifestations of TB-IRIS, though differences in TB-IRIS clinical manifestations can be explained by participants characteristics, such as severity of immunodeficiency and TB severity [20–22].

Although not directly explored in our study, in previous studies, timing of ART initiation after TB treatment was found to be a major risk factor for TB-IRIS, alongside with low CD4+ T-cell counts, high HIV-RNA, and extrapulmonary and disseminated TB [20–24]. Early ART initiation reduces all-cause mortality in PWH with CD4+ T-cell counts ≤ 50/mm³ but initiating ART early (<2 weeks) after TB treatment initiation increases the risk of TB-IRIS and death related to TB-IRIS, particularly among PWH with CD4+ T-cell counts ≤ 50/mm³ [25, 26]. In Reflate TB2, ART treatment was initiated at earliest 7 days and latest 57 days after TB treatment initiation and was not associated with the risk of TB-IRIS. We found that participants from Côte d’Ivoire and Vietnam were more likely to have TB-IRIS compared with those from Brazil and Mozambique; however, this association can be explained by lower CD4+ T-cells counts and higher HIV-RNA levels at ART initiation among participants from the former 2 countries [16].

In the Reflate TB-2 trial, virologic success rates (HIV-RNA <50 copies/mL) at weeks 4, 12, and 24 after ART initiation were 7%, 39%, and 58% in the efavirenz arm and 21%, 56%, and 58% in the raltegravir arm, respectively [16]. Despite that, we did not observe higher incidence of TB-IRIS in the raltegravir arm compared with the efavirenz arm. In the present analysis, virologic success rates were significantly lower at weeks 4, 12, and 24 after ART initiation in participants with TB-IRIS compared with those without TB-IRIS, and borderline significant at week 48, probably because of higher baseline HIV-RNA levels observed among those who developed TB-IRIS. On the other hand, TB treatment outcomes were not impacted by the occurrence of TB-IRIS, and proportions of bacteriological cure, treatment completion, and treatment failure were similar between the participants with and without TB-IRIS. Finally, death during TB treatment, by any cause, was observed more frequently in participants with TB-IRIS than in those without TB-IRIS but factors other than IRIS occurrence might explain this finding as participants with TB-IRIS were more severely ill. Advanced HIV and TB diseases, known risk factors for TB-IRIS, are also predictors of death by other causes, particularly from infections and other opportunistic illnesses. During the Reflate TB 2 study, 26 deaths occurred; the most common causes were opportunistic illnesses.

Our study has limitations. First, Reflate TB2 excluded PHW with TB meningitis, which probably explains our lower overall TB-IRIS incidence (10.6%) in comparison to a pooled estimate of 18% found in a metanalysis of 40 observational studies [5]. TB meningitis is associated with an 50% increased risk of TB-IRIS with poor outcomes, including mortality estimates ranging from 12% to 25% [10, 27–29]. Second, we found a lower than expected frequency of lymphadenopathy at TB treatment initiation, particularly hilar/mediastinal, that may be attributed to detection bias because a chest computed tomography scan was not required per protocol. Third, our study was not highly powered enough to assess differences in TB-IRIS mortality and other TB-IRIS outcomes by ART regimen. Fourth, TB-IRIS clinical management and investigation (including imaging studies) were at the discretion of local site investigators, though a standardized data collection was obtained timely from all IRIS cases. Finally, Reflate TB2 has strengths that should be highlighted. To date, Reflate TB 2 has included the largest study population, enrolled in 4 countries, in a comparative trial that evaluated INSTI versus NNRTI-based ART in PWH with TB and collected standardized data from all IRIS cases. To minimize the risk of misclassification bias, all reported IRIS cases were further revised/adjudicated by a central committee (ERC), according to INSHI criteria.

## CONCLUSION

Early introduction of INSTI-based ART in PWH treated for TB did not increase the incidence of TB IRIS compared with an efavirenz-based regimen. Advanced HIV disease, marked by low CD4+ T-cell counts and high HIV-1 RNA, as well as extrapulmonary and disseminated TB were associated with the occurrence of TB-IRIS. Second-generation INSTIs, especially dolutegravir, are now standard of care and pragmatic studies monitoring TB-IRIS in PWH treated with dolutegravir are desired to evaluate the incidence of TB IRIS in real-life settings and at a larger scale.

## Supplementary Material

ofae035_Supplementary_Data

## Supplementary Data

Supplementary materials are available at *Open Forum Infectious Diseases* online. Consisting of data provided by the authors to benefit the reader, the posted materials are not copyedited and are the sole responsibility of the authors, so questions or comments should be addressed to the corresponding author.

## Notes

***Acknowledgments*.** L. E. C., D. L., N. D. C., B. G., and C. C. conceived and designed the IRIS substudy. L. E. C., N. D. C., O. M., D. L., and B. G. contributed to the interpretation of the results. E. M., S. E., C. K., I. T., G. D. C., R. E., V. G. V., B. G., N. D. C., O. M., and J. M. M. implemented the trial and enrolled participants. C. C. did the statistical analysis. C. C. and L. E. C. prepared the report. L. E. C. wrote the first draft, and all authors reviewed and approved the final version of the report and the manuscript.

We thank all the participants who participated to the Reflate TB2 trial.

We thank the ANRS 12300 Reflate TB2 study group for their commitment to the trial.

We thank the national HIV and tuberculosis programs of participating countries for their support.

***Data sharing statement*.** Data from this study will not be shared publicly. Data could be made available by the sponsor (ANRS|MIE) to any researcher interested in getting the Reflate-TB2 data. Deidentified participant data would be made available with a data dictionary and shared under a data Transfer Agreement. Data can be requested at the following email address: nathalie.de-castro@aphp.fr.

***Financial support*.** The French National Agency for Research on AIDS and Viral Hepatitis and Emerging Infections (ANRS|MIE), Paris, France was the sponsor of the Reflate TB 2 trial. The Reflate TB2 trial was supported by the ANRS MIE (ANRS 12300), the Brazilian Ministry of Health in Brazil, and Merck Sharp Dohme-Chibret. The funders had no role in the analysis of the data nor in the manuscript writing.

## References

[1] Global Tuberculosis Report 2022 . 2023. Available at: https://www.who.int/teams/global-tuberculosis-programme/tb-reports/global-tuberculosis-report-2022. 10.1016/S2666-5247(22)00359-7 36521512

[2] World Health Organization . *Consolidated guidelines on HIV prevention, testing, treatment, service delivery and monitoring: recommendations for a public health approach*. Available at: https://apps.who.int/iris/handle/10665/342899. 34370423

[3] Dutertre M, Cuzin L, Demonchy E, et al Initiation of antiretroviral therapy containing integrase inhibitors increases the risk of IRIS requiring hospitalization. J Acquir Immune Defic Syndr 2017; 76:e23–6. 28418992 10.1097/QAI.0000000000001397

[4] Bonnet M, Baudin E, Jani IV, et al Incidence of paradoxical tuberculosis-associated immune reconstitution inflammatory syndrome and impact on patient outcome. PLoS One 2013; 8:e84585. 24367678 10.1371/journal.pone.0084585 PMC3867516

[5] Namale PE, Abdullahi LH, Fine S, Kamkuemah M, Wilkinson RJ, Meintjes G. Paradoxical TB-IRIS in HIV-infected adults: a systematic review and meta-analysis. Future Microbiol 2015; 10:1077–99. 26059627 10.2217/fmb.15.9

[6] Meintjes G, Lawn SD, Scano F, et al Tuberculosis-associated immune reconstitution inflammatory syndrome: case definitions for use in resource-limited settings. Lancet Infect Dis 2008; 8:516–23. 18652998 10.1016/S1473-3099(08)70184-1 PMC2804035

[7] Vignesh R, Balakrishnan P, Tan HY, et al Tuberculosis-associated immune reconstitution inflammatory syndrome—an extempore game of misfiring with defense arsenals. Pathogens 2023; 12:210. 36839482 10.3390/pathogens12020210 PMC9964757

[8] Walker NF, Stek C, Wasserman S, Wilkinson RJ, Meintjes G. The tuberculosis-associated immune reconstitution inflammatory syndrome: recent advances in clinical and pathogenesis research. Curr Opin HIV AIDS 2018; 13:512–21. 30124473 10.1097/COH.0000000000000502 PMC6181275

[9] Ravimohan S, Tamuhla N, Steenhoff AP, et al Immunological profiling of tuberculosis-associated immune reconstitution inflammatory syndrome and non-immune reconstitution inflammatory syndrome death in HIV-infected adults with pulmonary tuberculosis starting antiretroviral therapy: a prospective observational cohort study. Lancet Infect Dis 2015; 15:429–38. 25672566 10.1016/S1473-3099(15)70008-3 PMC4624391

[10] Pepper DJ, Marais S, Maartens G, et al Neurologic manifestations of paradoxical tuberculosis-associated immune reconstitution inflammatory syndrome: a case series. Clin Infect Dis 2009; 48:e96–107. 19405867 10.1086/598988

[11] Shelburne SA, Visnegarwala F, Darcourt J, et al Incidence and risk factors for immune reconstitution inflammatory syndrome during highly active antiretroviral therapy. AIDS 2005; 19:399–406. 15750393 10.1097/01.aids.0000161769.06158.8a

[12] Walker NF, Wilkinson KA, Meintjes G, et al Matrix degradation in human immunodeficiency virus type 1-associated Tuberculosis and Tuberculosis immune reconstitution inflammatory syndrome: a prospective observational study. Clin Infect Dis 2017; 65:121–32. 28475709 10.1093/cid/cix231 PMC5815569

[13] Tieu HV, Ananworanich J, Avihingsanon A, et al Immunologic markers as predictors of Tuberculosis-associated immune reconstitution inflammatory syndrome in HIV and tuberculosis coinfected persons in Thailand. AIDS Res Hum Retroviruses 2009; 25:1083–9. 19886838 10.1089/aid.2009.0055 PMC2828258

[14] Lennox JL, DeJesus E, Berger DS, et al Raltegravir versus efavirenz regimens in treatment-naive HIV-1–infected patients: 96-week efficacy, durability, subgroup, safety, and metabolic analyses. J Acquir Immune Defic Syndr 2010; 55:39–48. 20404738 10.1097/QAI.0b013e3181da1287 PMC6065510

[15] Psichogiou M, Basoulis D, Tsikala-Vafea M, Vlachos S, Kapelios CJ, Daikos GL. Integrase strand transfer inhibitors and the emergence of immune reconstitution inflammatory syndrome (IRIS). Curr HIV Res 2018; 15:405–10. 10.2174/1570162X15666171122155708 29173177

[16] De Castro N, Marcy O, Chazallon C, et al Standard dose raltegravir or efavirenz-based antiretroviral treatment for patients co-infected with HIV and tuberculosis (ANRS 12 300 Reflate TB 2): an open-label, non-inferiority, randomised, phase 3 trial. Lancet Infect Dis 2021; 21:813–22. 33667406 10.1016/S1473-3099(20)30869-0

[17] De Castro N, Chazallon C, N’takpe J-B, et al Determinants of antiretroviral treatment success and adherence in people with human immunodeficiency virus treated for tuberculosis. Open Forum Infect Dis 2022; 9:ofac628. 36540390 10.1093/ofid/ofac628 PMC9757687

[18] Zhao Y, Hohlfeld A, Namale P, Meintjes G, Maartens G, Engel ME. Risk of immune reconstitution inflammatory syndrome with integrase inhibitors versus other classes of antiretrovirals: a systematic review and meta-analysis of randomized trials. J Acquir Immune Defic Syndr 2022; 90:232–9. 35175970 10.1097/QAI.0000000000002937 PMC7612870

[19] Dooley KE, Kaplan R, Mwelase N, et al Dolutegravir-based antiretroviral therapy for patients coinfected with tuberculosis and human immunodeficiency virus: a multicenter, noncomparative, open-label, randomized trial. Clin Infect Dis 2020;70:549–56. 30918967 10.1093/cid/ciz256

[20] Abdool Karim SS, Naidoo K, Grobler A, et al Integration of antiretroviral therapy with tuberculosis treatment. N Engl J Med 2011; 365:1492–501. 22010915 10.1056/NEJMoa1014181 PMC3233684

[21] Havlir DV, Kendall MA, Ive P, et al Timing of antiretroviral therapy for HIV-1 infection and tuberculosis. N Engl J Med 2011; 365:1482–91. 22010914 10.1056/NEJMoa1013607 PMC3327101

[22] Blanc F-X, Sok T, Laureillard D, et al Earlier versus later start of antiretroviral therapy in HIV-infected adults with tuberculosis. N Engl J Med 2011; 365:1471–81. 22010913 10.1056/NEJMoa1013911 PMC4879711

[23] Laureillard D, Marcy O, Madec Y, et al Paradoxical tuberculosis-associated immune reconstitution inflammatory syndrome after early initiation of antiretroviral therapy in a randomized clinical trial. AIDS 2013; 27:2577–86. 24096631 10.1097/01.aids.0000432456.14099.c7

[24] Naidoo K, Yende-Zuma N, Padayatchi N, et al The immune reconstitution inflammatory syndrome after antiretroviral therapy initiation in patients with tuberculosis: findings from the SAPiT trial. Ann Intern Med 2012; 157:313. 22944873 10.7326/0003-4819-157-5-201209040-00004 PMC3534856

[25] Abay SM, Deribe K, Reda AA, et al The effect of early initiation of antiretroviral therapy in TB/HIV-coinfected patients: a systematic review and meta-analysis. J Int Assoc Provid AIDS Care 2015; 14:560–70. 26289343 10.1177/2325957415599210

[26] Uthman OA, Okwundu C, Gbenga K, et al Optimal timing of antiretroviral therapy initiation for HIV-infected adults with newly diagnosed pulmonary tuberculosis: a systematic review and meta-analysis. Ann Intern Med 2015; 163:32–9. 26148280 10.7326/M14-2979

[27] Marais S, Meintjes G, Pepper DJ, et al Frequency, severity, and prediction of tuberculous meningitis immune reconstitution inflammatory syndrome. Clin Infect Dis 2013; 56:450–60. 23097584 10.1093/cid/cis899 PMC3540040

[28] Agarwal U, Kumar A, Behera D, French MA, Price P. Tuberculosis associated immune reconstitution inflammatory syndrome in patients infected with HIV: meningitis a potentially life threatening manifestation. AIDS Res Ther 2012; 9:17. 22620862 10.1186/1742-6405-9-17 PMC3427137

[29] Bahr N, Boulware DR, Marais S, Scriven J, Wilkinson RJ, Meintjes G. Central nervous system immune reconstitution inflammatory syndrome. Curr Infect Dis Rep 2013; 15:583–93. 24173584 10.1007/s11908-013-0378-5 PMC3883050

