# Supplementary material for: Incidence and Predictors of Tuberculosis-associated IRIS in People With HIV Treated for Tuberculosis: Findings From Reflate TB2 Randomized Trial
Source: Open Forum Infect Dis. 2024 Jan 22;11(3):ofae035. doi: 10.1093/ofid/ofae035 (PMC10939434; doi:10.1093/ofid/ofae035)
Supplement: ofae035_Supplementary_Data [file ofae035_supplementary_data.docx]

Supplementary material

[TB-IRIS definition 1](#_Toc142233799)

[Table S1. Causes of death in participants of the Reflate TB-2 trial during the trial up to W48 2](#_Toc142233800)

TB-IRIS definition:

(Reference: Meintjes G, Lawn SD, Scano F, Maartens G, French MA, Worodria W, et al. Tuberculosis-associated immune reconstitution inflammatory syndrome: case definitions for use in resource-limited settings. *Lancet Infect Dis* 2008; 8:516–523.)

Cases of paradoxical TB-associated IRIS that fulfilled the three following components of the INSHI criteria were included:

(A) TB diagnosis was prior to ART initiation and initial response to TB treatment was observed; (B) clinical criteria including at least one major criterium (i.e., new or enlarging lymph nodes or cold abscesses, new or worsening radiological features of TB, new or worsening serositis) or two minor criteria (new or worsening constitutional symptoms [fever, night sweats or weight loss], new or worsening respiratory symptoms [cough, dyspnea, stridor], new or worsening abdominal pain [accompanied by peritonitis, hepatomegaly, splenomegaly, or abdominal adenopathy];

(C) absence of alternative explanations for clinical deterioration (i.e., TB drug resistance, poor adherence to TB treatment, another opportunistic illness, drug toxicity or reaction).

Cases with TB-IRIS manifestations onset up to 3 months after ART initiation were defined as **confirmed cases**.

Cases that fulfilled INSHI criteria but with first manifestations occurring between 3 and 6 months of ART initiation were considered as **probable cases**.

# Table S1. Causes of death in participants of the Reflate TB-2 trial during the trial up to W48

| **No.** | **Trial arm** | **Cause of death** |
| --- | --- | --- |
| 1 | Raltegravir | Neck injury |
| 2 | Raltegravir | Brain abscess |
| 3 | Raltegravir | Drug reaction with eosinophilia and systemic symptoms |
| 4 | Raltegravir | Unknown cause of death |
| 5 | Efavirenz | Sepsis |
| 6 | Efavirenz | Respiratory distress |
| 7 | Raltegravir | Unknown cause of death |
| 8 | Efavirenz | Sepsis |
| 9 | Raltegravir | Unknown cause of death |
| 10 | Raltegravir | Immune reconstitution inflammatory syndrome associated tuberculosis |
| 11 | Efavirenz | Cerebral toxoplasmosis |
| 12 | Raltegravir | Sepsis |
| 13 | Efavirenz | Acute renal failure |
| 14 | Efavirenz | Kaposi's sarcoma |
| 15 | Raltegravir | Atypical mycobacteria |
| 16 | Raltegravir | Meningoencephalitis |
| 17 | Efavirenz | Acute respiratory failure |
| 18 | Efavirenz | Unknown cause of death |
| 19 | Efavirenz | Atypical mycobacteria |
| 20 | Efavirenz | Hepatocellular carcinoma |
| 21 | Efavirenz | Immune reconstitution inflammatory syndrome associated tuberculosis |
| 22 | Efavirenz | Unknown cause of death |
| 23 | Raltegravir | Meningitis cryptococcal |
| 24 | Efavirenz | HIV wasting syndrome |
| 25 | Efavirenz | Disseminated tuberculosis |
| 26 | Raltegravir | Unknown cause of death |
